# Professionals' perspectives on loneliness and mental health among adolescents and emerging adults during educational transitions

**DOI:** 10.1080/17482631.2026.2743338

**Published:** 2026-11-06

**Authors:** Amanda Jasmin Emilia Sundqvist, Ottar Ness, Pia Nyman-Kurkiala, Jessica Hemberg

**Affiliations:** a Department of Natural and Health Sciences, Faculty of Science and Engineering, Åbo Akademi University, Vaasa, Finland; b Department of Education and Lifelong Learning, Faculty of Social and Educational Sciences, Norwegian University of Science and Technology (NTNU), Dragvoll, Norway

**Keywords:** Education, emerging adulthood, loneliness, mental health, transitions

## Abstract

**Introduction:**

Educational transitions alter academic demands, social relationships, and access to support. Yet less is known about how professionals interpret the conditions that shape loneliness and mental health across these transitions.

**Purpose:**

This study explored professionals’ accounts of factors that may intensify or mitigate loneliness and mental health challenges among adolescents and emerging adults during transitions to upper-secondary and higher education.

**Method:**

Four focus groups were conducted with 11 school psychologists, school counsellors, special education teachers, study supervisors, well-being coaches, project workers, and youth workers in Swedish-speaking regions of Finland. The interviews were analysed using reflexive thematic analysis.

**Results:**

Professionals described how individual circumstances and past adversity intersected with disrupted relationships and institutional arrangements. Transitions could offer autonomy and a fresh start, but fragmented peer groups, performance demands, weak continuity of support, and limited communication between institutions could heighten vulnerability. PParticipants emphasised early preparation, supportive teachers and peers, structured opportunities for social connection, and stronger collaboration between sending and receiving institutions.

**Conclusions:**

The findings position transition-related loneliness as arising through interactions between young people and the educational environments through which they move.

## Introduction

Loneliness is commonly described as the distressing experience that arises when a person’s social relationships are perceived as insufficient in quality or quantity (Kirwan et al., 2025; Perlman & Peplau, 1981). Adolescence and emerging adulthood are periods of heightened vulnerability. A recent meta-analysis estimated that 11.7% of adolescents aged 13–17 experience loneliness most or all of the time (Verity et al., 2025), while substantial levels have also been reported among emerging adults (Keiser et al., 2026). To date, most loneliness research focuses on individual predictors of loneliness, while less is known about how broader societal and contextual factors affect loneliness, such as the social environment, access to social services and support, or characteristics of surrounding environments (van den Berg et al., 2026).

Loneliness is shaped not only by individual circumstances but also by the relational and institutional contexts in which young people are located. Educational settings are especially important since they organize much of adolescents’ and emerging adults’ everyday social lives and provide access to peers, teachers, and support services (Jefferson et al., 2023; Verity et al., 2025). In Finland, pupils in primary and lower-secondary education and students in upper-secondary education are entitled to student welfare services, including school psychologists, social workers, and health services. These services are intended to operate as a preventive and multiprofessional whole involving education, health and social services, students, and families (Ministry of Education and Culture, n.d.; Ministry of Social Affairs & Health, 2026). Youth work and third-sector organizations may also contribute to local support networks, although their role and accessibility vary across settings. Entitlement to services does not establish how many professionals are available at each institution or how easy students find it to reach them; this study did not collect local staffing or access figures.

Support networks may nevertheless be disrupted when adolescents and emerging adults move between educational settings. Bronfenbrenner (1979) defined an ecological transition as a change in a person’s position within an environment following a change in role, setting, or both. In this study, educational transitions refer to expected progression from lower-secondary to upper-secondary education and from upper-secondary to higher education, rather than unplanned school changes, displacement, or migration. Expected transitions do not have uniform implications. Students who remain in the same locality may retain family and peer networks, whereas those who relocate must often establish new relationships while managing housing, finances, and distance from previous sources of support. Migration can add linguistic, cultural, and administrative demands, and community-based providers may therefore encounter needs that are not visible within educational institutions alone (Bessaha et al., 2023). Family responsibilities and mobility may also limit access to conventional student communities and complicate the negotiation of belonging (Mwale et al., 2018). The present study did not compare these groups directly; it examined how professionals interpreted such differences in their encounters with young people.

Educational transitions can unsettle established relationships, identities, and routines, while also creating opportunities for autonomy, new relationships, and a fresh start. A structure-agency perspective draws attention to how young people’s efforts to navigate change are enabled or constrained by their biographies, relationships, and educational arrangements (Rind, 2016). Loneliness during transition should therefore not be reduced either to an individual deficit or to a direct effect of institutional structures. It emerges through the interaction between young people and the environments through which they move.

Educational institutions are important settings for preventing loneliness and promoting mental health since they shape opportunities for participation, social connection, and access to support (Jefferson et al., 2023; Morrison & Peterson, 2013; Verity et al., 2025). Research on educational transitions has primarily examined students’ experiences (Hawkins et al., 2025; Sundqvist et al., 2024), although some studies include staff or community-provider perspectives (Bessaha et al., 2023; Cage et al., 2021). Less attention has been given to how professionals working across education, student welfare, and community services interpret the interaction of personal circumstances, relationships, and institutional conditions. Their position across settings may reveal how difficulties become visible, how support is coordinated, and where organizational discontinuities arise. This study addresses that gap.

## Aim and research questions

The study aimed to explore how professionals working with adolescents and emerging adults interpret loneliness and mental health in the school context and during transitions to upper-secondary and higher education. It also examined the strategies they considered relevant for preventing or mitigating difficulties. The research questions were: (1) How do professionals describe loneliness and mental health challenges in educational settings? (2) How do they interpret the relationship between educational transitions, loneliness, and mental health? (3) What strategies do they consider important for supporting young people during these transitions?

## Materials and methods

Purposeful and self-selection sampling were used. Purposeful sampling identifies participants with relevant knowledge or experience, while self-selection allows eligible participants to volunteer (Galloway, 2005; Palinkas et al., 2015). The Consolidated Criteria for Reporting Qualitative Research (COREQ) checklist guided reporting of the study (Tong et al., 2007).

Inclusion and exclusion criteria were established. The goal was to include professionals who work with adolescents and emerging adults aged 16–29 who had undergone an educational transition (transition to upper secondary/high school and/or the transition to higher education). Professionals such as school psychologists, school counsellors, special education teachers, well-being/school coaches, study supervisors, project workers, and youth workers, who had at least one year of work experience and worked with Swedish-speaking young people in Finland, were included. The goal was to include an equal number of women and men. Self-selection was implemented by allowing respondents to sign up for the interview on their own.

### Data collection and analysis

A semi-structured interview guide was utilized to collect the data. The initial questions were developed by the first author, and the second and fourth authors (JH, ON) reviewed them and offered critical feedback. The guide was refined as the interviews progressed. Open-ended questions were used to ensure the richness of the data. Example questions include: How do you view educational transitions in relation to loneliness and mental health? Is it possible that an educational transition can increase loneliness and decrease mental health? If so, how? Do you often meet young people who seek help because of problems related to increased loneliness and decreased mental health due to an educational transition? What kinds of problems do these young people describe? Are there risk factors in the school setting that can contribute to negative educational transitions? What would those be?

The first researcher contacted the managers of the organizations through email, provided details about the study and inquired whether they would be interested in participating. The managers either distributed information about the study to the workers or provided contact details to potential participants whom they thought would be a good fit for the study. The respondents were allowed to choose a suitable time for the interview. Participants were assigned to focus groups, which consisted of 2–4 professionals per group. A total of 4 focus groups were conducted, with 11 participants interviewed, consisting of 10 women and 1 man. The age range was 28–59 years, with a mean age of 45 years and a median age of 50 years. Their work experience varied from 1.5–13 years, their mean work experience was 6 years, and the median was 4 years. Seven participants worked with 16–19/20-year-olds, while four worked with 18–29-year-olds. For an overview of the participant sample, see Table 1.

**Table 1.** Focus group sample.

Focus group 1	Focus group 2	Focus group 3	Focus group 4
Youth worker Youth worker	School psychologist School psychologist	Special education teacher & study coach Project leader Study supervisor Well-being coach	Special education teacher & study supervisor School counsellor Professional coach

Sample adequacy was considered through information power rather than saturation (Malterud et al., 2016). This approach was chosen because reflexive thematic analysis does not assume that a finite set of themes can be exhausted through additional interviews (Braun & Clarke, 2021). The sample comprised professionals with direct experience of the phenomenon, the research questions were focused, and the interviews generated detailed accounts across educational and community settings. These features provided a sufficiently rich dataset for an in-depth analysis of the research questions, while not implying that further interviews could yield no additional perspectives.

### Data analysis

Reflexive thematic analysis (RTA) was selected since the study sought to develop an interpretive account of patterned meanings across professionals’ descriptions of loneliness, mental health, and educational transitions (Braun & Clarke, 2022). The analysis was informed by a contextualist epistemological position. Participants’ accounts were treated as situated professional interpretations rather than as direct representations of students’ lived experiences. RTA was appropriate because it allows attention to semantic and latent meanings and treats researcher subjectivity as an interpretive resource in theme development rather than as a source of bias to be eliminated (Braun & Clarke, 2022; Rind, 2021).

The analysis followed Braun and Clarke’s six recursive phases. AS led familiarisation, initial coding, and early theme development. JH and ON engaged with the transcripts and contributed alternative readings during reflexive analytic discussions, while PN-K participated in the further development and refinement of themes. These discussions were used to examine assumptions and consider competing interpretations, not to establish coding consensus or inter-rater reliability. AS maintained a reflexive log documenting analytic decisions and questions throughout the process. The analysis moved between data-led coding and interpretation of the individual, relational, and institutional conditions described by participants. No qualitative data-analysis software was used; coding and theme development were conducted manually in a shared working document.

### Ethical considerations

The guidelines of the Finnish National Board on Research Integrity TENK (2023) were followed. Participants received written and oral information about their rights, the study purpose and procedures, and their right to withdraw at any time. Interviews were audio-recorded and transcribed verbatim. Personal data were replaced with participant codes, and audio files were deleted after transcription. The ethics committee at Åbo Akademi University approved the study, and all participants provided written informed consent. Additional research permissions were obtained from the relevant organisations and authorities.

## Results

The analysis generated three overarching themes and seven subthemes (Table 2). The first theme describes how performance demands and school arrangements could limit opportunities for social connection. The second shows how a transition could offer a fresh start while also disrupting familiar relationships and support. The third concerns the support and collaboration professionals considered important for helping young people form relationships and maintain continuity across settings. Together, the themes show how young people’s circumstances and the settings they enter may shape their opportunities for connection and belonging.

**Table 2.** Overview of the three overarching themes and seven subthemes.

Overarching themes	Subthemes
Theme 1: Psychosocial strain in a performance-oriented youth culture	1.1 Navigating performance demands and expectations 1.2 School structures and developmental needs in conflict
Theme 2: Navigating turning points	2.1 Balancing growth and vulnerability 2.2 The role of past adversity in present adaptation
Theme 3: Increased need for organizational support and collaboration	3.1 Multiprofessional and network collaboration 3.2 Support in social, emotional, and academic matters 3.3 Early interventions and social activities

### Overarching theme 1. Psychosocial strain in a performance-oriented youth culture

The first theme links pressure to achieve with the ways schools organize learning and peer contact. In the first subtheme, participants described how academic demands and social comparison could leave less room for relationships, although paid work could also bring social contact. In the second, they considered how changing groups and independent study could make it more difficult to form dependable ties.

#### Subtheme 1.1: navigating performance demands and expectations

Participants described a performance-oriented youth culture in which academic demands, paid work, social comparison, and uncertainty about the future could accumulate. They observed that some upper-secondary students combined a workload resembling full-time study with part-time employment. Employment could add pressure, but it could also offer social contact, practical competence, and reassurance. This tension illustrates why the same circumstance could function as either a demand or a resource, depending on the young person’s situation and available support.

Participants linked distress to entrance examinations, frequent assessment, strong emphasis on grades, and expectations of early academic achievement. In their accounts, these demands could leave little time for rest and social life and could contribute to feelings of inadequacy, loneliness, and mental health difficulties.

*A third of our students work outside of school, and nowadays, high school studies are almost like a full-time job… So, this brings a certain level of stress, but it also provides social connections and a sense of entrepreneurship that can be reassuring and beneficial. It’s not all negative, but when it comes to the feeling of stress, it’s definitely a significant issue here, and we discuss it a lot. We talk about how to a learning process, and we also must work with the parents on this. How to manage it, and how to set boundaries, like saying, ‘I won’t take work shifts during exam week.’* (P7)

Participants interpreted social media as an arena that could intensify comparison and competition and reduce some opportunities for face-to-face interaction. They did not present digital media as a single cause of loneliness. Rather, they described how online comparison, academic expectations, and limited offline contact could combine in ways that made some young people more vulnerable.

*Through social media, people are somewhat influenced by American culture, where there is quite a lot of focus on achievement and ambition. And, and I don’t think that’s the main cause, but maybe one can start to see… fellow students more as competitors for the same jobs to a greater extent or compare one’s performance, which may then limit feelings of belonging, security, and relaxation in the sense that we are all here to socialize…* (R3)

Participants also associated difficulties in regulating smartphone use with distress and reduced opportunities for offline interaction. These observations reflected professional interpretations; the study did not examine young people’s digital practices directly.

#### Subtheme 1.2. School structures and developmental needs in conflict

Participants questioned whether school practices gave all students enough time and support to develop the skills expected of them. In their view, repeated experiences of falling short could affect how some young people saw themselves as learners. This was a professional interpretation of school life, not a finding from students’ own accounts.

*The way schools are structured today, all the way from elementary and middle school, does not support, to a sufficient degree… a sense of success for children and young people. Instead… skills are just supposed to be there from start, as if they should automatically exist before one has had the chance to practice them with support. If you think about Vygotsky’s zone of proximal development, then only a few children manage to cope with much of the learning the way it is structured today. So, I believe that within the structure, there is… a situation where many start to feel like failures quite early as students. Their self-image as learners in school is affected, which, in turn, creates mental health issues and… I don’t want to blame teachers at all, but there is something in this whole curriculum mindset that is not always based so much on research concerning brain development. As a result, many students, by the time they reach middle school, already feel like failures. Then comes the intense pressure…high demands, and one is expected to perform well… while the teaching style may only suit certain types of brains. And everyone else end up being labeled as losers, receiving diagnoses, struggling with anxiety… Too often, people look for the causes in the students’ home environments instead of examining the school environment itself.* (P4)

Participants were concerned that changing course groups in upper-secondary education gave some students little time to form stable ties. They linked assessment pressure and growing responsibility for independent study to strain, particularly when students had difficulty finding a group or an adult to turn to.

*I am also very critical of this lack of structure in high school, for example, and the fact that when you enter high school, you no longer have your own group in the same way. Instead, you take courses with different people all the time, so when it is most important to have that sense of belonging, that own group, and to receive feedback from your classmates, everything is too fragmented. And if you are a young person with social anxiety, this only increases it as a result… There is a lot in these school structures that just piles on more anxiety. There are so many presentations… sometimes you might have five presentations in a single week in high school. It becomes overwhelming, leading to burnout, so that by the time students reach university, they are already experiencing serious periods of burnout.* (P4)

Participants described how upper-secondary students were expected to make consequential subject choices early in their studies. They interpreted uncertainty about final examinations, further education, and future work as a source of pressure for some students. Similar uncertainty was described in higher education when students questioned their choice of field or had not entered their preferred programme.

*The academic pressure also seems to have increased from the moment students step into upper secondary/high school, needing to know which subjects to choose for the final exams… There seems to be a significant increase, perhaps happening three years earlier now than it did before. Previously, the focus was on preparing for entrance exams when applying to university, but now there is such a strong emphasis on the final grades (in upper secondary/high school), so this pressure starts earlier.* (P3)

Participants associated extensive independent study, large lectures, and limited continuity in peer groups with fewer opportunities for spontaneous contact. In their view, these arrangements could be isolating for students who had not already established relationships within their programme.

*I think many education programs, even without taking COVID-19 into account, have a lot of reading courses and self-studies, and many, many examination assignments… this can lead to loneliness if one hasn’t found a group of friends within their studies but would like to meet their classmates through their studies… But if the studies are very independent, there aren’t as many of those spontaneous meetings with people studying the same things, which one would need.* (P3)

Some participants mentioned that young people did not have an adult they could confide with regarding worries about loneliness. They connected this absence to limited time and availability among adults, while acknowledging that they mainly met young people who had already sought support.

*Most of the young people I meet are, in some way, quite lonely and really need to talk to a trustworthy person about whatever it may be. Many of them don’t really have anyone else to turn to, and I think the connection to mental health is very clear… this affects them significantly, making them feel much worse. They feel like they don’t have friends or any close family members to confide in, no one who asks them how they’re doing, how their school day was, or what they think and feel about different things.* (P8)

Participants distinguished chosen time alone from unwanted loneliness. They described young people who knew many peers but did not have someone with whom they could rely on, or speak openly with. In the same accounts, anxiety and stress often appeared alongside loneliness; however, the interviews cannot establish the direction of that relationship.

### Overarching theme 2. Navigating turning points

The second theme concerns what changes when young people enter a new setting. Its first subtheme describes greater autonomy and the chance to make new connections alongside uncertainty and separation from established networks. The second describes how previous exclusion and other circumstances could affect the prospect of joining new groups. Professionals described different possibilities, not a single pattern for all students.

#### Subtheme 2.1. Balancing growth and vulnerability

Professionals generally viewed the transition to upper-secondary education as positive for many students. They described a calmer environment, greater autonomy, opportunities to concentrate on chosen subjects, and the possibility of a fresh start. For others, unfamiliar expectations and a heavier workload could produce early strain. The accounts therefore presented transition as a variable turning point rather than an inherently negative event.

*For many, it’s a chance to start over at a new school. So, for the most part, it’s a positive change, both in terms of loneliness and mental health. Moving from a chaotic lower secondary school, to a high school where they can focus on their studies in a calmer environment…it’s a different experience. So, I would say that 9 out of 10 are positive about the transition.* (P10)

Professionals described anxiety, uncertainty, stress, and anticipation among some young people approaching higher education. Their accounts linked these responses to forming new friendships, adapting to unfamiliar staff and teaching practices, possible relocation, finances, and separation from established support networks.

*Fear and uncertainty about change…But also this sense of wonder or questioning… ‘How will I manage when high school is over, and I must move on?‘ In a way, it’s a fear… because there, I won’t have my usual network of contacts that I’m used to here. And I won’t have the support from teachers and study counselors. I feel that for many, that uncertainty really comes up.* (P11)

#### Subtheme 2.2. The role of past adversity in present adaptation

Participants interpreted previous bullying and other adverse social experiences as shaping expectations of new peer environments. A transition could offer a fresh start, but fear of repeated exclusion could make group placement and social participation especially demanding.

*Previous experiences of bullying that they are very afraid might happen again… There is a lot of stress about… who they will end up in a group with, and for example, even though they don’t have all their lessons with their own group, it’s still something they think about a lot in advance…* (R8)

Participants identified several circumstances that could heighten vulnerability, including difficulties interpreting social cues, ADHD, social anxiety, school-attendance difficulties, depression, and prior loneliness. These categories were not treated as determining outcomes. Rather, professionals described how they could interact with unfamiliar environments, limited support, and demands for rapid social and academic adaptation.

*Those dealing with social anxiety, bullying, and then NEPSY, the students with NEPSY-related difficulties… It’s like they are the ones who often struggle with these things (transitions)… and thosewho have depression, deep depression.* (R4)

Participants also described students with children or other family responsibilities, students who relocated, and those who did not participate in dominant forms of student social life. These circumstances could narrow opportunities to meet peers with similar experiences and make belonging more difficult, although the study did not compare these groups systematically.

### Overarching theme 3. Increased need for organizational support and collaboration

The third theme concerns the support professionals thought could help young people form and maintain connections across educational settings. Its subthemes cover coordination between services, relationships with teachers and peers, and early preparation and opportunities to meet others. These are participants’ proposals; the study did not test their effectiveness.

#### Subtheme 3.1. Multiprofessional and network collaboration

Participants wanted clearer contact between schools, student welfare services, youth workers, and community groups. They saw a risk that support would fall between services when nobody took responsibility for making contact. One participant also argued that links to leisure activities might reach young people who were less involved in school-based activities.

*I think we should also involve associations, the third sector, and youth activities related to leisure, because it’s all connected. Young people spend time together outside of school as well. And these sports clubs engage many of our young people… There’s a great opportunity to build on values like tolerance and equality there…So, if we can establish cooperation beyond the school environment, we can also reach homes and leisure activities…and especially those young people who don’t participate in any activities and are at risk of dropping out, the ones who just hang around town… How do we reach them?* (P10)

Participants identified discontinuity in information and support as a particular institutional concern. They described how confidentiality requirements, reliance on disclosure by students or guardians, and staff turnover could delay the recognition of support needs. These accounts did not imply that confidentiality should be weakened; they pointed instead to the need for lawful, consent-based planning and clearer communication between sending and receiving institutions.

*One risk factor is that school counselors, psychologists, and those conducting assessments have confidentiality obligations and belong to the welfare sector… We don’t receive any information from them…they are not allowed to share it with us…So, if we need any information regarding an assessment or any psychological matter, it must come directly from the guardians or the student themselves, as the counselor, psychologist, or doctor is allowed to provide it only to them. This can create a break in the chain… Even though we remind guardians that the only way we can receive this information is through them or the student, there is still a risk that it doesn’t get passed on.* (P9)

#### Subtheme 3.2. Support in social, emotional, and academic matters

Participants saw teachers and tutors as important points of contact for new students. One suggestion was to let teachers assign groups for several weeks so that students would not have to find partners on their own on the first day. They also wanted room for different ways of taking part in school life, including for students who did not seek out large social events.

*The teacher starts by selecting the groups themselves at first, so that students don’t have to find a group on their own and end up thinking, ‘Now I’m alone again.’ Instead, the teacher decides, and then you stay in the same group for maybe four weeks. That creates a sense of security, and after that, you might feel ready to start practicing switching groups on your own.* (P4)

Participants stressed the value of teachers or counsellors who had time to listen and could help students find the right support. They suggested speaking with students about the coming transition early enough to plan for difficulties, while also asking what the young person wanted from the new setting. In their accounts, practical help with workload and dependable adult relationships were connected.

*Well, in a school context, when it comes to mental health, it’s often about stress… about managing feelings of stress and handling a heavy workload… Upper secondary studies are quite demanding nowadays…So, as educators, it’s our job to work with these young people, to give them the tools they need, and to help them recognize their strengths. That way, even if they experience a dip in their mental health, they still have strengths to rely on and work from.* (P7)

#### Subtheme 3.3. Early interventions and social activities

Participants saw it as important for students to visit the receiving institution prior to transitioning to the institution. One suggestion was to spend more time in a prospective study programme, so that students could get to know its teaching and daily routines. They also noted that late admission decisions could leave little time to plan a move or arrange support.

*For those finishing ninth grade, before they start thinking about what they want to do when they grow up, they should have the chance to explore their options…not just for a few hours, but by choosing one or two study programs and spending one, two, or even three days there. That way, they can really see what it’s like and what the program includes.* (P2)

Some participants argued for responding to difficulties before the school change, rather than waiting until a student struggled in the new setting. This included talking about mental health and agreeing, with the young person, how existing support might continue.

*I really think that the foundation needs to be laid as early as middle school. We need to start working on mental health there because if that is in order then, the transitions won’t be as difficult either.* (P2)

Participants mentioned study groups, after-school activities, school camps, and common rooms as places where students might meet others. In their accounts, such opportunities were part of the educational setting, alongside more direct support from teachers and peers. The interviews did not establish who used these places or whether particular arrangements reduced loneliness.

## Discussion

This study examined how professionals interpreted loneliness and mental health in educational settings and during transitions to upper-secondary and higher education, and what forms of support they considered important. Its contribution lies less in identifying previously unknown risk factors than in showing how personal circumstances, relationships, and institutional arrangements become connected, accordring to professional accounts. From this perspective, transition-related loneliness is not located solely within the individual. Instead, it develops through encounters between the individual, the relationships they encounter, and the educational environments through which they move.

The results support a multilevel interpretation. Individual’s prior experiences, and personal resources shaped how change was approached; relational continuity influenced young people’s social networks and support systems; and institutional arrangements organized access to peers, adults, and support. These levels were interdependent. For example, prior bullying could make entry into a fragmented peer environment especially difficult, while structured group-building and an accessible teacher could alter the same transition. This interaction is summarized in Table 3.

**Table 3.** Multilevel interpretation of the findings.

Analytical level	Conditions in professionals’ accounts	Interpretive implication
Individual	Prior bullying, anxiety, ADHD, coping resources, uncertainty, finances, and family responsibilities	Transitions may offer autonomy and a fresh start or heighten strain, depending on biography and available resources.
Relational	Continuity or disruption of peer, family, teacher, and community relationships	Belonging depends on opportunities to maintain or establish meaningful relationships and access trusted adults.
Institutional	Course organization, assessment demands, self-directed study, access to support, confidentiality, staff turnover, and inter-institutional communication	Organizational arrangements distribute opportunities for social connection, recognition, and timely support.

At the individual level, professionals described variation rather than a uniform transition effect. Increased autonomy and a new environment could be liberating and offer a fresh start, while uncertainty, performance demands, financial pressures, and previous adversity could heighten strain. A structure-agency perspective helps explain this variation: young people act within educational and social conditions that both enable and constrain their efforts to adapt (Rind, 2016). Accounts of students with family responsibilities or mobility-related challenges similarly show how biography and institutional expectations may conflict (Mwale et al., 2018). These findings do not establish that one type of transition is generally more psychologically demanding than another.

At the relational level, participants emphasised the loss, continuity, and reconstruction of everyday ties. Peers, families, teachers, tutors, and other trusted adults could provide practical help and a sense of being recognised. Conversely, unstable groups, separation from established networks, and limited access to adults could contribute to loneliness. Previous research likewise identifies peer and family support, group-building activities, and inclusive educational climates as important during transitions (Jaud et al., 2023; Jefferson et al., 2023; Norris & Mounts, 2010; Stokoe et al., 2024; Zhang, 2026). Students who relocate or migrate may need to rebuild several forms of support at once, making coordination with community-based services particularly relevant (Bessaha et al., 2023). In the European Loneliness Survey, the association between perceived support and lower loneliness was stronger than for contact frequency; the analysis also found regional differences in the association with digital contact (Yenihayat et al., 2026). These results helps distinguish frequency of contact, from feeling supported and connected to others. Though the survey was not confined to students, it is likely that similar associations are present among students.

At the institutional level, the professionals drew attention to course organization, assessment pressure, extensive self-directed study, confidentiality procedures, staff turnover, and communication between sending and receiving institutions. These arrangements shaped when students met peers, whether support needs became visible, and whether assistance continued across settings. Rind and Kadiwal (2016) similarly show that educational practices are formed within institutional structures rather than through individual choices alone. The distinctive contribution of the present study is to show how professionals located loneliness partly in these organisational conditions, while also recognising that their effects depended solely on young people’s relationships and circumstances.

The professionals’ cross-setting perspective also revealed tensions in support. Confidentiality protects students’ rights, yet participants described uncertainty about how necessary support could be prepared without relying entirely on disclosure after a transition. Early planning should therefore be consent-based and specific about what information is needed, who may receive it, and how the student participates. Continuity also depends on stable staffing, clear responsibilities, and accessible points of contact. These are organisational questions, not simply matters of individual help-seeking.

The proposed measures may be difficult to implement. Earlier admission decisions, transition visits, structured social activities, regular check-ins, and stronger inter-institutional collaboration require time, staffing, coordination, and clarity about responsibility. Young people may also hesitate to seek help because of stigma, uncertainty about services, or concern about how others will respond (Eigenhuis et al., 2021). Evidence from school-based prevention and university transition research likewise points to the need for coordinated approaches that extend beyond individual interventions (Caldwell et al., 2019; Worsley et al., 2021). Schools and higher-education institutions should therefore combine universal measures that create opportunities for connection with targeted, low-threshold support. Teachers and tutors need sufficient time to notice changes and establish trust, while school welfare services and community actors need workable routes for referral and collaboration. A scoping review of the built environment and loneliness describes how accessible places may shape opportunities for encounter and how people feel in those places (van den Berg et al., 2026). This gives a reason to examine the common rooms and other informal meeting places mentioned by participants, without assuming that providing a space alone will reduce loneliness.

The results should be read as professionals’ situated interpretations, not as direct evidence of students’ experiences or causal effects. Their accounts nevertheless identify points at which personal resources, relationships, and institutional practices meet. Future studies should examine these processes with students and professionals, compare transitions with and without relocation, and follow changes in belonging and support over time. Future work could also ask young people which shared spaces they actually use and whether those spaces make it easier to meet others or to feel at ease (van den Berg et al., 2026).

## Strengths and limitations

A strength of the study is the inclusion of professionals from several fields and educational settings. Their accounts made it possible to examine how difficulties and support needs are interpreted across organizational boundaries. At the same time, the data represent professional observations rather than young people’s lived experiences. The analysis therefore cannot establish causal relationships or determine how common the described processes are.

Zoom enabled participation across geographical areas and provided scheduling flexibility. Online focus groups may, however, alter turn-taking, non-verbal communication, and spontaneous interaction (Archibald et al., 2019). The study did not systematically record technical disruptions or compare online and in-person interaction, so it is not possible to determine how the format shaped the material.

Although the recruitment aimed for gender balance, self-selection resulted in a sample of 10 women and one man. Research has identified gender differences in preferences for psychological help and coping (Liddon et al., 2018), but the present material cannot show whether participant gender influenced the accounts, reflects the gender composition of the professions, or affected which students sought support. The small sample and its concentration to Swedish-speaking services also limit transferability to other parts of Finland and to different educational systems. The sample was adequate for the focused interpretive analysis, but further interviews could have generated other perspectives.

## Conclusion

Professionals described educational transitions as variable turning points whose implications depended on the interaction of individual circumstances, relationships, and institutional arrangements. A new setting could provide autonomy, acceptance, and a fresh start. It could also intensify loneliness and distress when previous adversity, disrupted networks, performance demands, fragmented peer groups, or weak continuity of support converged. The findings therefore caution against treating transition-related loneliness as an individual problem or as the direct result of a single institutional factor. Educational institutions can respond by preparing students early, organizing inclusive opportunities for sustained peer contact, ensuring access to trusted adults and welfare services, and strengthening consent-based coordination between sending and receiving institutions. These measures require stable responsibilities, time, and collaboration across education, health, youth work, and community services. Future research should include young people’s own accounts, distinguish transitions involving relocation or migration, and examine how belonging and support change over time.

## Data Availability

Because of ethical and confidentiality restrictions, the supporting qualitative data are not publicly available.

## Generative Artificial Intelligence (AI) statements

During revision of this manuscript, M365 Copilot and ChatGPT were used for language editing and readability. The authors critically reviewed and revised all suggested text, verified the cited sources, and take full responsibility for the content of the manuscript.
